# 非供者特异性HLA抗体对异基因造血干细胞移植临床结局的影响

**DOI:** 10.3760/cma.j.cn121090-20251218-00601

**Published:** 2026-08

**Authors:** 瑾宁 杨, 晗 肖, 昕 李

**Affiliations:** 中南大学湘雅三医院血液内科，长沙 410013 Department of Hematology, the Third Xiangya Hospital, Central South University, Changsha 410013, China

## Abstract

回顾性分析2016年1月至2025年7月期间在中南大学湘雅三医院接受异基因造血干细胞移植（allo-HSCT）且完成了抗HLA抗体筛查患者的临床资料，探讨非供者特异性HLA抗体（NDSA）对allo-HSCT临床结局的影响及去敏治疗的潜在价值。共379例患者接受allo-HSCT且完成了抗HLA抗体筛查。排除自体移植或非本中心完成移植者、DSA阳性者及缺少用于判定抗体性质与供受者配对的核心信息者，最终纳入281例（83.6％）抗HLA抗体阴性患者及55例（16.4％）NDSA阳性患者。根据抗体平均荧光强度（MFI）将NDSA阳性患者分为1 000≤MFI<10 000组32例（58.2％）和MFI≥10 000组23例（41.8％）。与抗HLA抗体阴性组相比，NDSA阳性组患者年龄更大、女性及有妊娠史者比例更高（均*P*<0.05）。在未接受抗体去敏的全相合移植患者中，NDSA阳性组3年总生存（OS）率显著低于抗体阴性组（*P*＝0.043）。多因素Cox回归分析示NDSA MFI≥10 000是全相合allo-HSCT患者3年OS与无事件生存（EFS）的独立危险因素（OS：*HR*＝4.364，95％ *CI*: 1.041～18.291，*P*＝0.044；EFS：*HR*＝3.453，95％ *CI*: 1.035～11.518，*P*＝0.044）。倾向评分匹配后，去敏组的3年OS率［100％对（66.7±15.7）％，*P*＝0.123］与EFS率［（83.3±15.2）％对（53.3±17.3）％，*P*＝0.253］均高于未去敏组，但差异均无统计学意义。上述结果表明NDSA MFI≥10 000可能是影响全相合allo-HSCT患者长期生存的危险因素，针对这类患者进行抗体去敏治疗有望改善其远期预后。

## Abstract (en)

We retrospectively analyzed the data of patients who underwent allogeneic hematopoietic stem cell transplantation（allo-HSCT）and completed anti-HLA antibody screening at our center between January 2016 to July 2025 to examine the impact of non-donor specific HLA antibodies（NDSA）on the clinical outcomes in this patient population and to analyze the potential efficacy of using desensitization therapies. Among the 618 patients screened patients, 379 underwent allo-HSCT. Fifty-five patients were NDSA-positive. Based on the screening results, NDSA-positive patients were divided into the following groups according to the mean fluorescence intensity（MFI）: 1 000≤MFI<10,000 and MFI≥10,000 groups. Compared with the antibody-negative group, the NDSA-positive patients were older, more likely to be female, and had a higher rate of prior pregnancy（*P*<0.05）. In the fully matched transplants without desensitization, the 3-year overall survival（OS）was significantly lower in the NDSA-positive group（*P*＝0.043）. Stratified analysis showed that NDSA MFI of ≥10,000 was as an independent risk factor for inferior 3-year OS（*HR*＝4.364, 95％ *CI*: 1.041–18.291, *P*<0.05）and event-free survival（EFS）（*HR*＝3.453, 95％ *CI*: 1.035–11.518, *P*<0.05）. In the propensity score-matched NDSA-positive cohort, the desensitization group showed numerically higher 3-year OS［100％ *vs*（66.7±15.7）％, *P*＝0.123］and EFS［（83.3±15.2）％ *vs*（53.3±17.3）％, *P*＝0.253］than the untreated group. Our study findings suggest that NDSA MFI of ≥10,000 may be associated with poorer long-term survival in patients receiving fully matched allo-HSCT. Desensitization therapy may improve the long-term outcomes of these patients.

异基因造血干细胞移植（allo-HSCT）是治疗血液系统恶性肿瘤的重要方式，移植前采用高灵敏度方法进行抗人类白细胞抗原（HLA）抗体筛查已逐渐成为常规评估移植风险、指导供者选择的重要环节[1]–[2]。根据受者体内预先存在的抗HLA抗体与供者HLA抗原的对应关系，可将其分为供者特异性抗体（DSA）及非供者特异性HLA抗体（NDSA）两类。中高滴度DSA是导致移植物排斥、植入延迟、移植物功能不良及生存率下降的独立危险因素[3]，且其检测流程的规范化、结果解读及去敏治疗方式已形成专家共识[1],[4]–[6]。

NDSA不直接针对供者的HLA抗原，其是否会影响移植患者预后以及是否需在移植前进行清除仍存在争议。部分研究认为NDSA的存在未能显著影响移植结局[7]–[14]，主动使用血浆置换清除抗体未能带来获益[8]。但也有报道揭示了NDSA的存在可能与中性粒细胞植入延迟、巨细胞病毒（CMV）再激活及输血次数增加相关[8]–[11],[15]。这一争议提示需要进一步探索NDSA在allo-HSCT中的意义。本研究回顾性分析NDSA及其不同抗体滴度水平对移植预后的影响，并探索抗体去敏治疗的潜在价值，现报道如下。

## 病例与方法

一、病例

本研究为单中心、回顾性、观察性队列研究，收集2016年1月至2025年7月期间在本中心接受allo-HSCT并完成了抗HLA抗体筛查的所有患者的临床资料，包括人口统计学特征、移植特征、NDSA状态、是否接受抗体去敏治疗、输血次数和量、是否发生移植物抗宿主病（GVHD）、生存结局及末次随访时间等。所有纳入患者在移植前均有完整的临床资料及抗体筛查结果，排除经检测存在DSA的患者。所有患者均接受了标准预处理方案，按方案类型分为阿糖胞苷联合白消安/环磷酰胺（Ara-C+BuCy）和非Ara-C+BuCy两类。非Ara-C+BuCy方案包括塞替派联合白消安/环磷酰胺/氟达拉滨（TBC/TBF）及其他个体化预处理方案。具体方案的选择依据原发病、患者状况及移植中心规范确定。

二、抗HLA抗体的检测与去敏治疗

通过移植前评估阶段患者血清样本检测预先存在的抗HLA抗体。采用Luminex 200流式点阵仪，使用LABScreen Mixed混合试剂进行混合抗体筛查并确定其对应的平均荧光强度（MFI），筛查阳性样本进一步采用LABScreen Singles明确抗体特异性，并与供者HLA分型结果对比以判定是否为NDSA。根据实验室标准，混合抗体检测MFI≥1 000被判定为阳性。基于全相合NDSA阳性患者MFI分布特征（*P*_50_＝9 479.15）及临床可操作性，本研究选取10 000作为分层界值，将抗体阳性患者分为1 000≤MFI<10 000组和MFI≥10 000组。抗体去敏治疗方法包括血浆置换、免疫吸附、利妥昔单抗中的一种或多种，并联合静脉注射免疫球蛋白，具体方案由临床医师及患者结合具体病情共同决定。根据是否接受去敏治疗将全相合NDSA阳性患者分为去敏组和未去敏组。

三、观察指标

中性粒细胞植入定义为移植后连续3 d中性粒细胞绝对值≥0.5×10^9^/L的第1天。血小板植入为连续7 d无需输注血小板支持下，血小板计数≥20×10^9^/L。GVHD根据国际共识标准诊断。病毒再激活定义为移植后100 d内出现CMV-DNA或EBV-DNA病毒载量>400拷贝/ml。总生存（OS）期定义为从移植日至任何原因导致死亡或末次随访的时间。无事件生存（EFS）期为从移植日至首次发生疾病进展、复发或因任何原因死亡的时间。随访信息来自住院和门诊病历以及电话随访记录。随访截止日期为2025年10月，中位随访时间为42.6（95％*CI*: 36.7～47.7）个月。

四、统计学处理

使用SPSS 26.0进行统计分析，绘图使用GraphPad Prism 9软件。分类变量以例（％）表示，计量资料使用*M*（范围）或*x*±*s*描述。计量资料比较采用*t*检验或秩和检验，率的比较采用卡方检验或Fisher精确检验。采用Kaplan-Meier法绘制生存曲线，并用Log-rank检验进行组间比较。采用反向Kaplan-Meier法估算中位随访时间。多因素生存分析采用Cox比例风险模型。为平衡去敏治疗相关的混杂因素，在全相合NDSA阳性患者中以是否接受去敏治疗为标准进行了1∶1倾向评分匹配，匹配变量包括年龄、性别、抗体MFI水平，卡钳值为0.2，后续比较均基于此匹配队列。双侧*P*<0.05为差异具有统计学意义。

## 结果

一、病例特征

共618例患者完成了抗HLA抗体的筛查，总体抗体阳性率为38.3％（237/618）。其中379例患者接受了造血干细胞移植，79例（20.8％）患者为抗HLA抗体阳性。排除自体移植或非本中心完成移植者、DSA阳性者及缺少用于判定抗体性质与供受者配对的核心信息者，最终纳入281例抗HLA抗体阴性患者及55例（16.4％）NDSA阳性患者。根据NDSA MFI水平，分为1 000≤MFI<10 000组32例（58.2％）和MFI≥10 000组23例（41.8％）。与抗HLA抗体阴性组相比，NDSA阳性组的患者年龄较大，女性及有妊娠史者比例更高，全相合供者和外周血干细胞来源比例更高。两组在原发病、预处理方案、输血史及移植前疾病状态等方面差异均无统计学意义（均*P*>0.05）。筛选流程及详细基线特征见附图1和附表1（**扫描本文首页二维码查阅**）。

在未接受抗体去敏治疗的患者中，与抗HLA抗体阴性患者相比，NDSA阳性患者的OS差异无统计学意义（*HR*＝0.99，95％ *CI*: 0.45～2.18，*P*＝0.975），供者类型的交互作用检验呈边缘显著（交互作用*P*＝0.051），因此后续分析进一步聚焦于全相合移植人群（**扫描本文首页二维码查阅附图2**）。

二、NDSA阳性与抗HLA抗体阴性患者的特征及结局比较

在全相合移植患者中，比较未接受抗体去敏治疗NDSA阳性组（24例）与抗HLA抗体阴性组患者（91例，1例在随访起点前死亡因此未纳入临床结局分析）的基线特征。两组患者在植入时间、输血需求、住院时间、病毒再激活及GVHD发生率等方面差异均无统计学意义（均*P*>0.05，**扫描本文首页二维码查阅附表2**）。在EFS与OS方面，抗HLA抗体阴性组的3年OS率显著高于NDSA阳性组［（91.0±3.3）％对（69.3±10.7）％，*P*＝0.043］，而3年EFS率差异无统计学意义（图1）。两组1年、2年OS及EFS率均无显著组间差异。

**图1:**
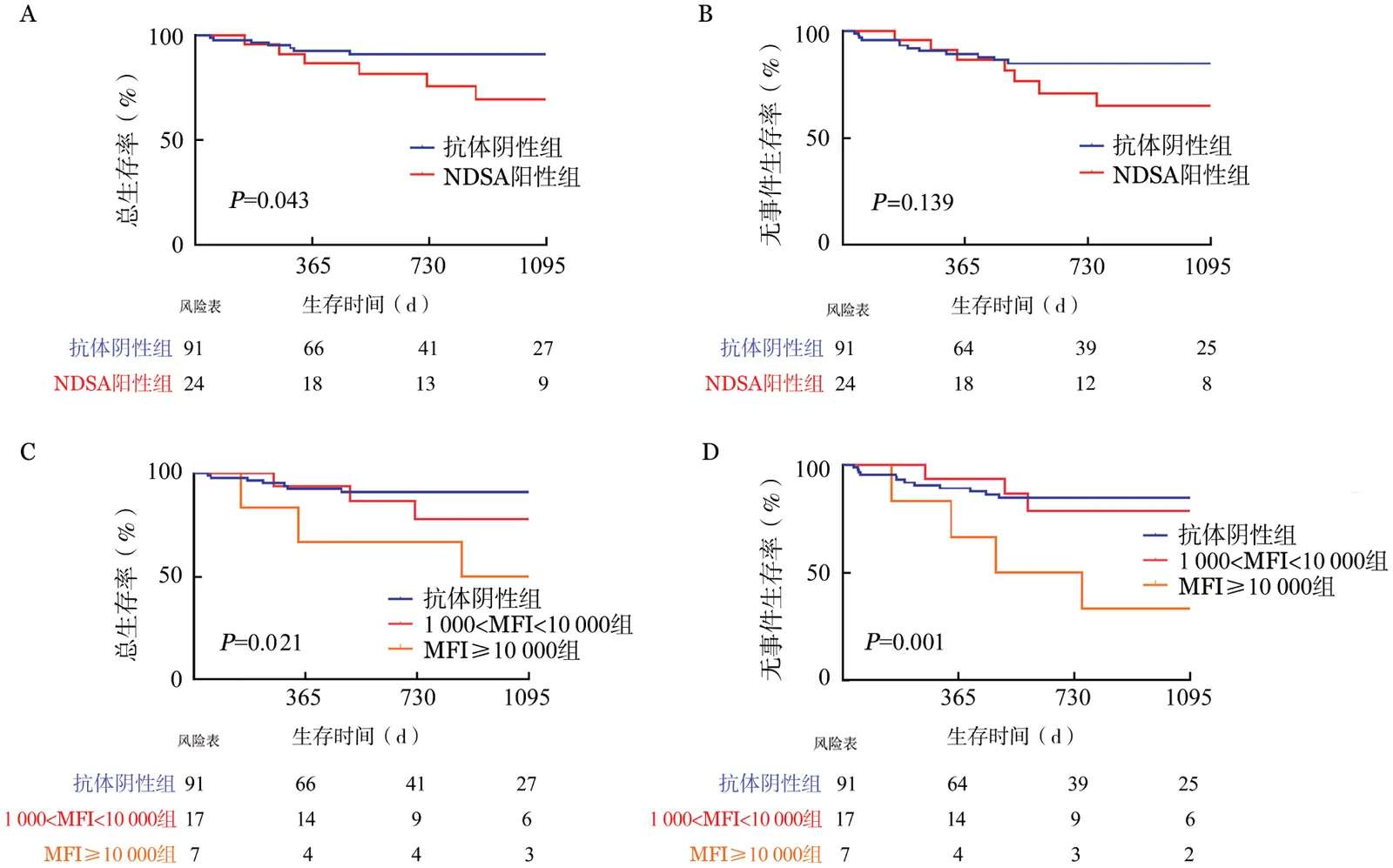
全相合allo-HSCT患者NDSA阳性组与抗HLA抗体阴性组的OS（A）和EFS（B），以及不同抗体滴度水平组的OS（C）和EFS（D）曲线 **注** allo-HSCT：异基因造血干细胞移植；NDSA：非供者特异性HLA抗体；OS：总生存；EFS：无事件生存；MFI：抗体平均荧光强度

三、NDSA滴度与预后的关系

在全相合移植患者中，阴性组（91例）、1 000≤MFI<10 000组（17例）、MFI≥10 000组（7例）3年OS率与EFS率差异均有统计学意义（OS：*P*＝0.021；EFS：*P*＝0.011），MFI≥10 000组预后最差（图1）。多因素分析显示，MFI≥10 000是影响3年OS（*HR*＝4.364，95％ *CI*: 1.041～18.291，*P*＝0.044）与EFS（*HR*＝3.453，95％ *CI:* 1.035～11.518，*P*＝0.044）的独立危险因素（表1）。但由于该组样本量较小（7例）且效应估计的95％*CI*较宽，宜谨慎解读结果。

**表1:** 全相合allo-HSCT患者OS及EFS的单因素及多因素Cox分析

因素	OS						EFS					
	单因素分析			多因素分析			单因素分析			多因素分析		
	*HR*	95％ *CI*	*P*值	*HR*	95％ *CI*	*P*值	*HR*	95％ *CI*	*P*值	*HR*	95％ *CI*	*P*值
年龄≥35岁	0.583	0.190～1.782	0.344	0.606	0.193～1.901	0.390	0.553	0.210～1.354	0.186	0.585	0.225～1.518	0.270
女性	2.211	0.681～7.184	0.187	1.688	0.487～5.953	0.409	2.119	0.805～5.578	0.128	1.761	0.632～4.906	0.279
非恶性血液系统疾病	1.262	0.280～5.698	0.762				0.767	0.177～3.323	0.723			
预处理非Ara-C+BuCy	1.691	0.568～5.034	0.346				1.592	0.646～3.920	0.312			
移植前未达CR	0.898	0.323～2.493	0.836				1.061	0.509～2.210	0.875			
MFI水平												
阴性组	参照						参照					
1 000≤MFI<10 000组	1.983	0.513～7.674	0.321	1.896	0.472～7.619	0.367	1.126	0.318～3.993	0.854	1.074	0.294～3.917	0.914
MFI≥10 000组	5.624	1.450～21.805	0.012	4.364	1.041～18.291	0.044	4.725	1.522～14.667	0.007	3.453	1.035～11.518	0.044

**注** allo-HSCT：异基因造血干细胞移植；OS：总生存；EFS：无事件生存；Ara-C：阿糖胞苷；BuCy：白消安+环磷酰胺；CR：完全缓解；MFI：抗体平均荧光强度

四、抗体去敏治疗对预后的影响

为了评估抗体去敏治疗在NDSA阳性患者中的疗效，我们比较了去敏组与未去敏组的基线及移植特征（**扫描本文首页二维码查阅附表3**），发现两组在年龄、性别及抗体水平等关键因素上差异有统计学意义。为控制上述混杂因素，我们以上述变量进行了1∶1倾向评分匹配分析，最终纳入20例患者。匹配后两组粒系植入时间、输血需求、GVHD发生及病毒激活等早期临床结局未观察到明确差异（均*P*>0.05），去敏组的血小板植入时间较未去敏组有缩短趋势［11（9～15）d对13（10～23）d，*P*＝0.066］（**扫描本文首页二维码查阅附表4**）。抗体去敏组3年的OS率［100％对（66.7±15.7）％］和EFS率［（83.3±15.2）％对（53.3±17.3）％］均高于未去敏组，但差异均无统计学意义（OS：*P*＝0.123；EFS：*P*＝0.253，图2）。

**图2:**
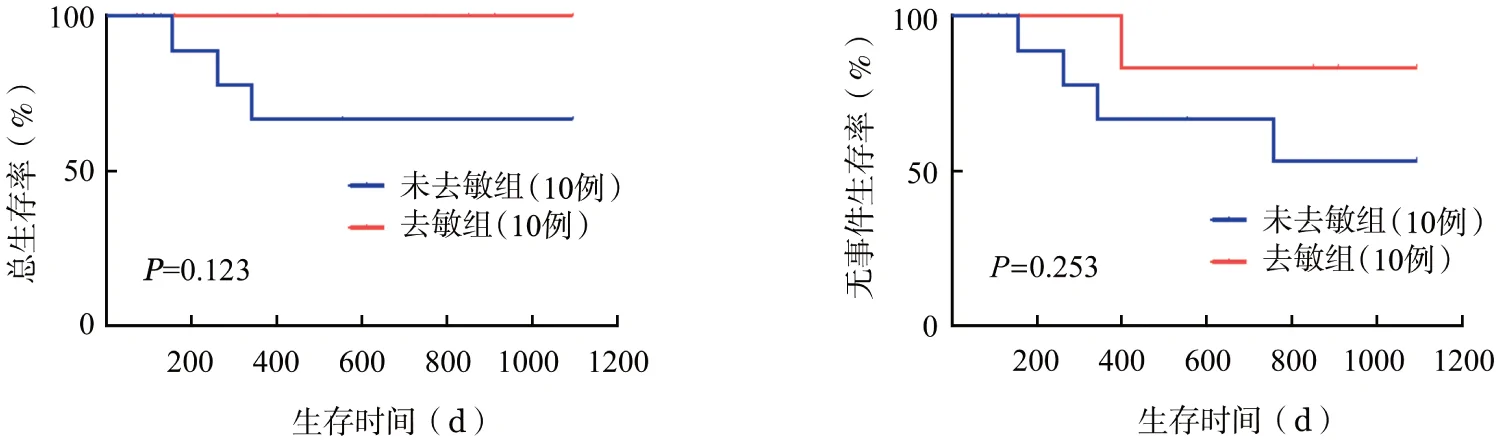
经倾向评分匹配后抗体去敏治疗组与未去敏组患者的3年生存结局比较

## 讨论

本研究通过回顾性分析本中心接受造血干细胞移植且完成抗HLA抗体筛查患者资料，发现在全相合移植患者中，NDSA MFI≥10 000可能对患者的长期生存产生不利影响，针对抗HLA抗体的去敏治疗有望改善患者的预后。

我们的数据显示NDSA阳性患者多为女性、年龄较大且多有妊娠史（*P*<0.001），与既往研究一致[16]–[17]。此前也有研究指出，抗HLA抗体的产生还与输血、较长病程、初诊时血小板计数及疾病类型等因素相关[18]–[19]。我们还发现NDSA阳性患者更常接受全相合移植（*P*<0.001）。考虑到供者选择偏好可能影响对NDSA的预后评估，我们进行了亚组及交互作用分析，结果显示NDSA对生存的影响可能因供者类型的不同而存在差异。这一发现与既往研究一致，即供者类型是影响患者预后的关键因素之一[20]。Detrait等[21]在血液系统恶性肿瘤患者的研究中发现，抗HLA抗体阳性患者的3年OS率显著降低（16％对34％）。Milano等[11]报道，在HLA不全相合移植患者中NDSA阳性的患者中位OS期仅为21个月，明显短于抗体阴性组的61个月（*P*＝0.01）。Wei等[10]发现在全相合移植患者中NDSA阳性与移植早期OS率下降有关（*P*＝0.012）。在此基础上，本研究在全相合移植患者中发现NDSA阳性组的3年OS率显著低于阴性组（*P*＝0.043），进一步证实了NDSA在这一特定人群中的不良预后价值。不同研究之间的结果差异可能与研究人群的基线特征、抗体滴度阈值的界定以及各中心诊疗策略的不同等因素相关。

NDSA的预后影响可能与抗体水平有关。多因素Cox回归分析显示，MFI≥10 000的NDSA是影响OS及EFS的独立危险因素。不同的研究采用的抗体滴度界值及其临床结局存在差异，可能源于各实验室在检测方法与结果判读标准等方面的不一致。在此基础上，本研究进一步评估了抗体去敏治疗能否改善这类患者的预后。倾向评分匹配分析后，结果显示接受抗体去敏治疗的患者较未去敏者具有更优的生存结局趋势。Ersoy等[8]在地中海贫血患者中的研究显示，抗体去敏治疗未显著影响植入时间、输血需求或其他移植结果，但本研究中观察到了生存曲线分离趋势，提示抗体去敏治疗在NDSA MFI≥10 000的患者中具有潜在价值，这一发现值得进一步验证。

NDSA MFI≥10 000影响长期生存结局机制尚未明确。受供受者HLA分型分辨率与抗体检测范围的限制，部分与供者相关的抗体可能未被识别，其潜在机制可能与DSA相似。抗HLA抗体可与供者造血干细胞及免疫细胞表面HLA结合，通过补体途径、抗体依赖性细胞毒作用或巨噬细胞的吞噬作用等引发供者细胞损伤，进而增加植入失败、移植物功能不良或免疫重建延迟的风险[22]。这些早期事件可能会通过增加感染、非复发死亡等风险进而影响长期生存。既往研究也提示抗体强度及补体结合能力与风险程度可能相关[23]。但NDSA与不良预后相关的机制可能与DSA存在不同之处，未来仍需进一步探索。

综上所述，本研究提示NDSA MFI≥10 000是全相合移植患者长期生存的危险因素，针对此类患者进行抗体去敏治疗可能改善远期生存。但本研究为回顾性、观察性队列研究，样本量有限，虽采用多因素分析和倾向评分匹配控制混杂，但仍无法完全避免未测量或残余混杂的影响，限制了因果推断。未来还需进一步阐明NDSA导致不良预后的具体机制，并通过开展更大规模的前瞻性研究验证针对这一高危人群的最佳干预策略及疗效。

## Supplementary Material


